# Preference for concentric orientations in the mouse superior colliculus

**DOI:** 10.1038/ncomms7773

**Published:** 2015-04-02

**Authors:** Mehran Ahmadlou, J Alexander Heimel

**Affiliations:** 1 Netherlands Institute for Neuroscience, an institute of the Royal Academy of Arts and Sciences, Cortical Structure & Function group, Meibergdreef 47, 1105 BA Amsterdam, The Netherlands

## Abstract

The superior colliculus is a layered structure important for body- and gaze-orienting responses. Its superficial layer is, next to the lateral geniculate nucleus, the second major target of retinal ganglion axons and is retinotopically organized. Here we show that in the mouse there is also a precise organization of orientation preference. In columns perpendicular to the tectal surface, neurons respond to the same visual location and prefer gratings of the same orientation. Calcium imaging and extracellular recording revealed that the preferred grating varies with retinotopic location, and is oriented parallel to the concentric circle around the centre of vision through the receptive field. This implies that not all orientations are equally represented across the visual field. This makes the superior colliculus different from visual cortex and unsuitable for translation-invariant object recognition and suggests that visual stimuli might have different behavioural consequences depending on their retinotopic location.

## Abstract (web summary)

The mammalian superior colliculus (SC) processes visual stimuli but little is known about the spatial organization of the response preferences for specific visual features. Here the authors show that the mouse SC contains a map for orientation preference such that preferred grating orientation is aligned to concentric circles around the centre of the visual field.

## Graphical abstract

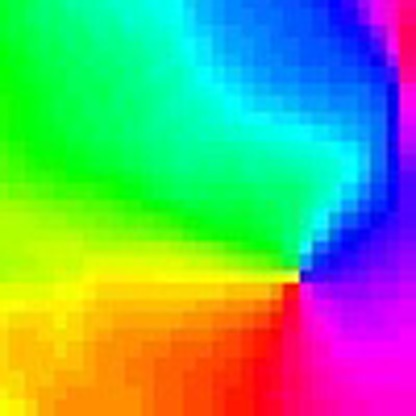

Cells in the primary visual cortex (V1) respond optimally to edges or lines of a particular orientation. This is widely regarded as an essential step in visual processing and object recognition. In carnivores and primates, orientation tuning is organized such that cells within a vertical column of V1 generally prefer the same orientation^1^. Rodents lack this organization of their visual cortex^2^,^3^. The thalamocortical pathway, however, is not the only major visual pathway in rodents. In mice, a majority of retinal ganglion cells send projections to the superficial layer of the superior colliculus (sSC)^4^. The sSC in rodents, primates and carnivores is retinotopically organized and almost exclusively visual in its responses. The deeper layers of the superior colliculus (SC) are multimodal, and show responses to visual, somatic and auditory stimuli. The SC contains eye and body-centred topographic representations of visual, auditory and somatic space^5^,^6^. These topographic maps are in remarkable register across layers, such that in one column perpendicular to the tectal surface, cells respond to different modalities providing sensory input to approximately the same parts of space. The primary role of the SC is to use this information to initiate downstream motor programmes directing the sensory organs of the head towards objects of interest^7^. In primates, the SC is particularly essential for the direction of gaze, and its virtual form of attention^8^,^9^.

The rodent SC, on the other hand, does not only play a role in orienting, but is also involved in avoidance and escape behaviour^10^ and spatial navigation^11^. Compared with primates, it is relatively large in comparison with visual cortex and therefore a larger role for the SC in visual processing is suspected in the rodent^12^. As circumstantial evidence for this, most neurons in the rodent sSC do not respond indiscriminately to any visual activity in their receptive field, but are selective to orientation^13^,^14^,^15^. This has not been observed in primates and carnivores, although some direction selectivity is present in the sSC of all these species^15^. An organization of orientation preferences has not been reported in the sSC of any species. Certainly in the mouse sSC, one would expect a lack of spatial organization, because there is not even clustering of orientation preference in the rodent visual cortex^2^,^3^. The recent discovery, however, that in mouse the axons of several groups of direction-selective retinal ganglion cells terminate in the sSC in a patchy manner^16^,^17^ suggests that there may be more organization of feature preference in the rodent sSC than previously recognized^18^. For this reason, we were curious to investigate the functional organization in the rodent sSC. Using a combination of extracellular electrophysiology and wide-field calcium imaging, we found that responses are not only organized retinotopically, but also by orientation preference. Orthogonal to the surface, neurons respond preferrentially to the same retinotopic location and the same orientation. Neurons most often have a preferred orientation parallel to the concentric circle around the centre of vision through their receptive field. This preference is there for both static and drifting gratings. Our finding implies that not all orientations are represented equally for all visual field locations, unlike the representation of preferred orientations in the mammalian visual cortex.

## Results

### Columnar organization of orientation

**Figure 1:**
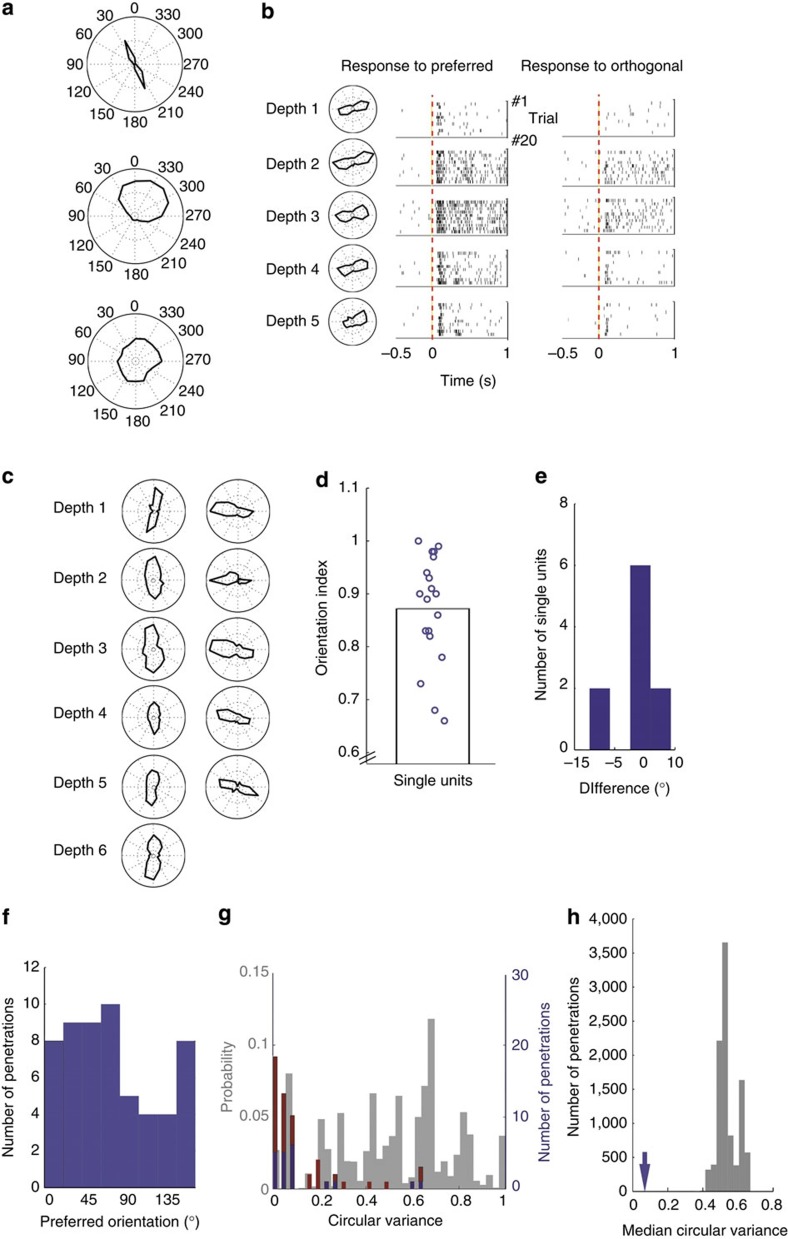
Vertical grouping of orientation preference in the superficial layer of the mouse superior colliculus. (**a**) Examples of orientation and direction tuning. (**b**) Orientation tuning across different channels on a laminar electrode with 50 μm spacing. Right panels show raster plot of spikes around the onset of all trials to the preferred and orthogonal orientations. (**c**) Example penetrations with tungsten microelectrodes, depths are 50 μm apart. (**d**) Orientation selectivity index of 19 high waveform-amplitude single units from horizontal and vertical penetrations (9 mice). Bar indicates mean. (**e**) Difference of the preferred orientation of the 10 single units (5 mice) encountered on vertical penetrations to the mean preferred angle of all units on the penetration. (**f**) Histogram of the circular mean of the orientation preference for all 57 penetrations (14 mice). (**g**) Histogram of the circular variance of orientation preference in individual penetrations with a silicon probe (blue, *n*=20) or tungsten microelectrode (red, *n*=37), plotted on the probability distribution generated by shuffling all recorded preferences over all penetrations (grey). (**h**) Median circular variance for the real penetrations (arrow) lies far below medians for shuffled data.

We recorded the responses to full-screen square-wave drifting gratings in the sSC using extracellular recordings in anaesthetized mice. In agreement with earlier findings^5^,^15^, we found cells that are highly selective for orientation or direction and cells that respond indiscriminately to all directions (Fig. 1a). We made electrode penetrations using linear silicon electrodes inserted less than 15° from perpendicular to the tectal surface. On most penetrations, single and multi-units recorded at different depths preferred the same orientation, suggesting a columnar organization (Fig. 1b). This was surprising as orientation columns had not been reported in the sSC or optic tectum of other species. Furthermore, previous investigations, notably by Hubel who co-discovered orientation columns in the cat and primate visual cortex, did not find such columns in the mouse sSC^5^. Our findings with the silicon probes were confirmed by tungsten microelectrodes recordings (Fig. 1c). In one penetration, we encountered enough single-units to confirm our finding with single-units alone. The orientation selectivity and the alignment of orientation preferences on the other penetrations was not an effect of combining the spikes of multiple neurons, as the mean orientation selectivity for the single units that we recorded in the sSC was higher than that of the combination of multi- and single-units (mean Orientation Index (OI) single-units=0.87, *n*=19, Fig. 1d, mean OI all units=0.49, *n*=270, *P*<0.001, Mann–Whitney *U*-test). Also, the orientation preference of the ten single-units that were recorded in any of the perpendicular penetrations with the high-impedance tungsten microelectrodes matched the mean preferred orientation of the complete penetration (Fig. 1e, see also Supplementary Fig. 1 for an example single unit). The same orientation preference continued until about 300 μm deep, where a clear drop in visual response per unit (from about 9 Hz to 2 Hz, *P*<10^−7^, Mann–Whitney *U*-test) indicated the border between the superior and intermediate layers of the SC.

One trivial way that vertical clustering of orientation would be observed is if there were a strong orientation bias across the whole sSC. However, a previous study did not find such a bias^15^. We also found all orientations almost equally represented across all 57 penetrations (*P*=0.11, Rayleigh test for circular non-uniformity, 14 mice, Fig. 1f). On 14 of our penetrations, we did not find a consistent orientation selectivity. To determine whether the low spread in orientation preference in the other penetrations could be due to chance, we computed the circular variance of preferred orientation for all penetrations and compared these to the distribution that we got when we 10,000x reshuffled the preferences of all the 270 recorded units over all penetrations (Fig. 1g). The median circular variance over the penetrations for all shuffles was higher than the real median, making it unlikely that the low spread in orientation preferences occurred by chance (*P*<0.0001, Fig. 1h). Supplementary Fig. 2a shows that the vertical similarity of orientation preference, as indicated by a low circular variance of preference across a penetration, is smaller at low and high spatial frequencies, but over the entire range of spatial frequencies, it is not significantly different within the bounds of our statistical power (*P*=0.2, Kruskal–Wallis, 10 penetrations, 6 mice). The mean orientation index per penetration is different across the full range of spatial frequencies (*P*=0.0003, Kruskal–Wallis, 10 penetrations, 6 mice, Supplementary Fig. 2a), reflecting that the orientation selectivity is lower below 0.04 cycles per degree (c.p.d.), but there is no significant difference for the OI from 0.04 to 0.4 c.p.d. (*P*=0.12, analysis of variance (ANOVA), 10 penetrations, 6 mice). The preferred orientation is very similar when measured at either 0.08 and 0.16 c.p.d., or 0.1 and 0.2 c.p.d. (Supplementary Fig. 2b). The vertical similarity of orientation preference also does not change over a large range of temporal frequencies and contrasts (Supplementary Fig. 3).

### Organized independently of V1

**Figure 2:**
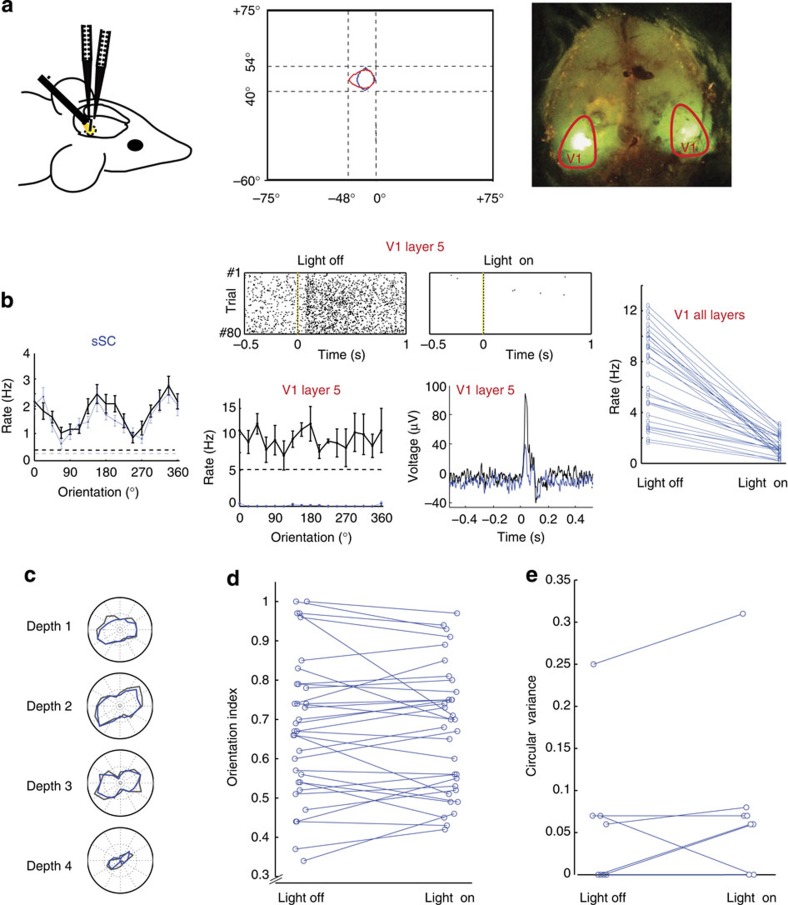
Vertical grouping of orientation is independent of visual cortex. (**a**) Two recording probes are placed in matching retinotopic positions in V1 and the sSC, whereas a LED-coupled fibre was placed above V1. The middle panel shows the ON-response fields for one V1 site (red) and sSC site (blue) during a simultaneous recording. The right panel is an example of expression by viral transfection imaged by fluorescent microscope. The red lines indicate the estimated outline of V1 based on stereotactic coordinates. (**b**) Responses of multi-units in sSC and layer 5 of binocular V1 in the injected area of an example experiment (left and middle-down left) and visual evoked potential in layer 5 of V1 (middle-down right) during light-off condition (black) and light-on conditions (blue) show that V1 L5 is silenced, while sSC remains active. Error bars in tuning curves are mean±s.e.m. Time 0 s is the onset of the visual stimulus. The light was turned on 1 s before this. The middle-top panel is the corresponding raster plot of spikes in V1 L5 when the light is off (left) and on (right). The right panel shows the effect of shining light on silencing all layers of V1 of 6 mice (*n*=29). (**c**) Example tuning curves from four depths in the sSC, separated by 50 μm, with V1 active (black) and silenced (blue). (**d**) Orientation tuning of units with V1 active (left) and silenced (right) is not changed (*P*=0.99, Wilcoxon signed rank sum test, *n*=33, 6 mice). (**e**) Circular variances of eight vertical penetrations in the sSC in light off (V1 active) and light on (V1 silenced) conditions is not significantly changed (*P*=0.31, Wilcoxon signed rank sum test, 6 mice).

To check whether the orientation tuning and its vertical organization were dependent on the extensive connections from visual cortex to sSC^7^,^19^, we optogenetically silenced primary visual cortex using an archaerhodopsin variant expressed by transfection of an AAV virus injected at five depths in up to three locations. We recorded simultaneously in V1 and the sSC at retinotopically matching positions (Fig. 2a). Shining light on the visual cortex strongly reduced activity in V1, whereas activity levels in the sSC were not much affected (Fig. 2b). With reduced activity in the visual cortex, the vertical grouping of orientation preference was as clear as it was with an active cortex (Fig. 2c) and orientation selectivity was not reduced (*P*=0.99, Wilcoxon signed rank sum test, *n*=33, 6 mice, Fig. 2d). This finding is in agreement with a report that orientation selectivity in the mouse sSC is not reduced when visual cortex is aspirated^15^. The mean circular variance along the penetrations was also not affected by silencing V1 optogenetically (*P*=0.31, Wilcoxon signed rank sum test, *n*=8, 6 mice, Fig. 2e). The same results were obtained by silencing V1 with muscimol (Supplementary Fig. 4). These findings indicate that in mature mice the vertical organization of orientation selectivity in the sSC is independent of cortical input.

### Horizontal organization

**Figure 3:**
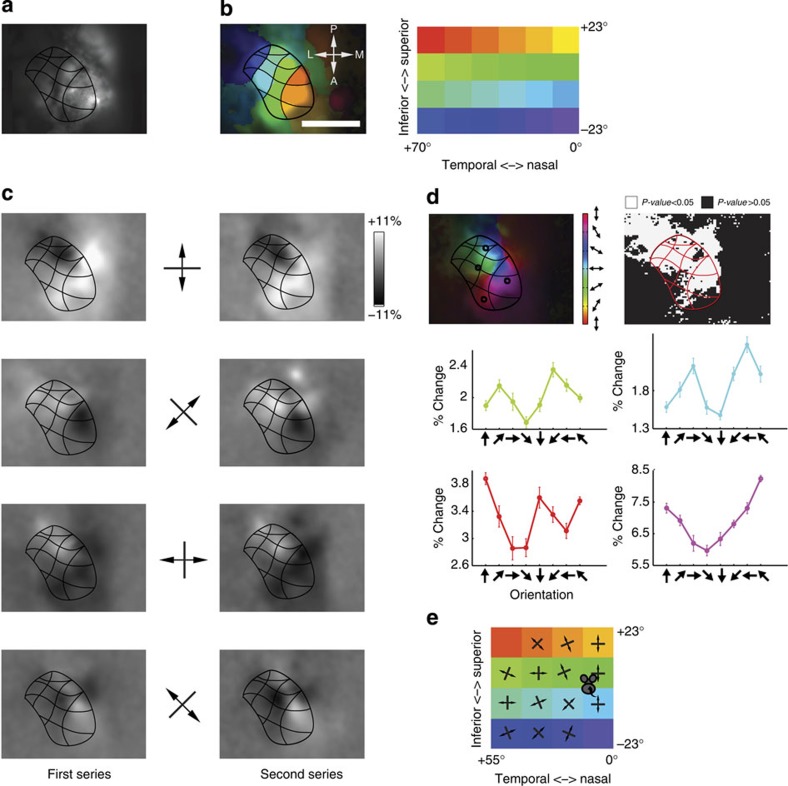
Orientation preference is dependent on retinotopic position. (**a**) Image of GCaMP6 fluorescence in the sSC after neocortex has locally been aspirated. (**b**) Retinotopy in sSC. Each pixel is coloured corresponding to the monitor patch to which it gave most response. Pixel saturation scales linearly with response strength. Maximum Δ*F*/*F* was 8%. A smooth mesh was drawn over the boundaries of the monitor patch representations. This mesh was added to the other panels only as a visual aid. Scale bar is 1 mm. (**c**) The difference of the average response for each set of orientations and the average response to all orientations shows regional differences, which are very consistent for the first set of 10 (left) and next set of 10 presentations (right). (**d**) Left top panel shows polar map of sSC, where each pixel is coloured to its angular preference. Saturation scales linearly with response strength. Maximum Δ*F*/*F* was 7%. The map shows a smooth change covering all angles once. For the four indicated locations (each disk with a radius of 40 μm, covering area of 40 pixels) at different corners of the responding region, the response to all directions are shown at the bottom. Error bars in tuning curves are mean±s.e.m. Right top panel shows the significance of the preference of each sSC pixel for any of the four orientations. *P*-values of the white pixels are less than 0.05. (**e**) For each patch in the retinotopy of **b**, the preferred orientation is computed and shown on the equivalent part in visual space. This reveals a preference for concentric orientations.

To understand the organization of orientation preference along the tectal surface, we made a number of horizontal penetrations. These showed a remarkably slow change in optimal preferences. Rate of change was about 10° per 100 μm with an occasional sharper turn (Supplementary Fig. 5). However, using this method it was difficult to develop an overall impression of the horizontal mapping of orientation. Therefore, we decided to also do macroscopic imaging of calcium responses^20^ to oriented gratings after careful suction of the overlying cortex. Retinotopic mapping showed that we imaged 10–45% of the total sSC surface (7 mice, Fig. 3a,b, and Supplementary Figs 6a and 7). Next, we showed full-screen gratings drifting along the horizontal, vertical and major oblique directions. The visible sSC responded strongly to all orientations (Supplementary Fig. 6b), but there were clear regional differences in the responses to the different orientations, which were very consistent across trials (Fig. 3c). Computation of a polar vector map where the hue of each pixel represents its average orientation, revealed a smooth map (Fig. 3d), which was very different from the orientation pin-wheel map known from the visual cortex of other mammals^21^,^22^. Instead, a single smooth circular progression through all angles is laid out over the retinotopic map. The responses at sample locations (disks with a 40-μm radius) revealed that the polar map gives a good representation of the orientation preferences (Fig. 3d), although at the more anterior-medial visible part of the sSC, there was a clear preference for upward motion, rather than a preference for horizontal orientation (Fig. 3d, bottom right).

### Preference for concentric orientations

When we combined the retinotopy and orientation imaging, and plotted the computed preferred orientation for each part of the represented visual space (from the retinotopy in Fig. 3b), the structure of the organization became more apparent (Fig. 3e). What had not been directly apparent from our initial extracellular recordings was that each column in the sSC responds to a specific retinotopic location and prefers gratings oriented perpendicular to the radial angle with respect to the nose of the animal, or, in other words, parallel to the concentric circle around the centre of vision at the receptive field location. In the small representation of the very nasal ipsilateral visual hemifield that is present in the sSC, there was a preference for horizontal orientation. It is clear from this representation that, unlike the pinwheel orientation maps in carnivores and primates, there is no even representation of all preferred angles for each point of the visual space in the sSC.

**Figure 4:**
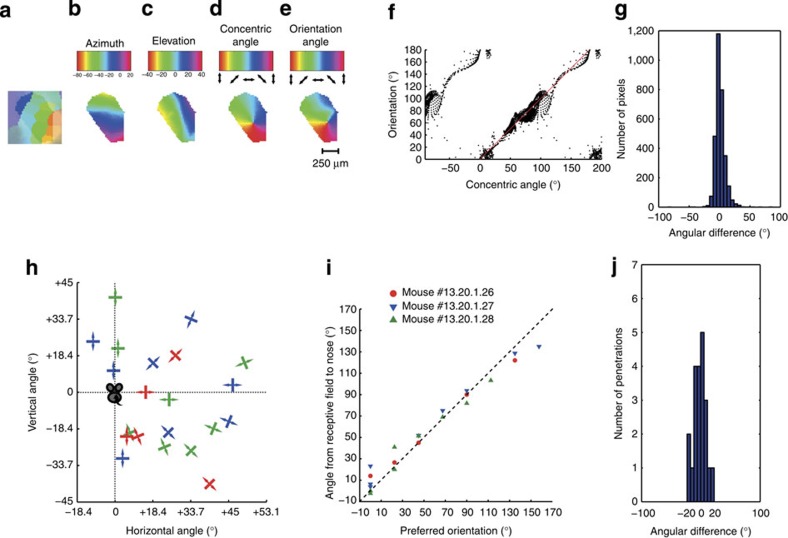
Concentric orientation preferences. (**a**) Retinotopic map of one example mouse captured by wide-field calcium imaging. The area inside the computed convex hull of the centre responses for all monitor patches is highlighted and used in the following analyses. (**b**,**c**) The retinotopic map of **a** recomputed in azimuth and elevation coordinates, respectively. (**d**) Map of the concentric angle (that is, the angle orthogonal to the radial angle for the centre of vision) reconstructed based on **b** and **c**. (**e**) The corresponding imaged orientation map. (**f**) Preferred orientation of the pixels of orientation maps versus the concentric angles computed from the retinotopy for all mice (*n*=7). (**g**) Histogram of angular difference between preferred orientation and computed concentric angles of the pixels (7 mice). (**h**) Preferred orientation of penetrations (*n*=21, 3 mice), obtained by extracellular recordings, shown at the corresponding receptive field positions. Different colours indicate different mice. (**i**) Concentric angles at receptive fields relative to the nose versus the corresponding preferred orientations based on the data shown in **h**. Different colours indicate different mice. (**j**) Histogram of angular difference between preferred orientation and concentric angles of units shown in **h** and **i**.

We quantified this preference for concentric orientations by interpolating the representation of azimuth and elevation with respect to the nose from the retinotopic map (Fig. 4a–c) and calculating for each imaged pixel the concentric angle, that is, the orientation perpendicular to the radial angle of the represented visual space. For the nasal ipsilateral representation, we selected the horizontal orientation. When we convolved this map with a two-dimensional (2D) Gaussian to simulate the effects of retinotopic and light scatter, we obtained a map that was remarkably similar to the measured map for preferred orientation (Fig. 4d,e, see also partial maps in Supplementary Fig. 7). For the combination of the pixels of the maps from all mice, the measured preferred orientation was highly correlated to the inferred concentric orientation (circular correlation coefficient=0.92, *P*<10^−5^, 7 mice, Fig. 4f) and the difference of preferred orientation and concentric angle (for pixels of the contralateral visual field) was centred close to zero (Fig. 4g). The imaged orientation preferences were similar for different spatial frequencies and insensitive to a 180° change in spatial phase. Supplementary Fig. 8a shows the vector angle difference of each pixel in response to drifting gratings with a difference of 180° in their initial phase (1 mouse) and Supplementary Fig. 8b shows the vector angle difference of each pixel in response to drifting gratings with two different spatial frequencies (two mice). When we used the same recording protocol as Fig. 4 for the visual cortex, no consistent functional organization beyond retinotopy became apparent (Supplementary Fig. 9) and no pixels had a significant (for *α*=0.05) preference for any orientation.

We made a number of electrode penetrations in the sSC (21 penetrations, 3 mice) to confirm the layout of this concentric orientation map (Fig. 4h). Like the imaged maps, the measured preferred orientation was almost identical to the concentric orientation (corr. coef=0.98, *P*<10^−5^, Fig. 4i) and the difference of preferred orientation and concentric angle was centred close to zero (Fig. 4j).

### Tuning for orientation, not motion-axis

**Figure 5:**
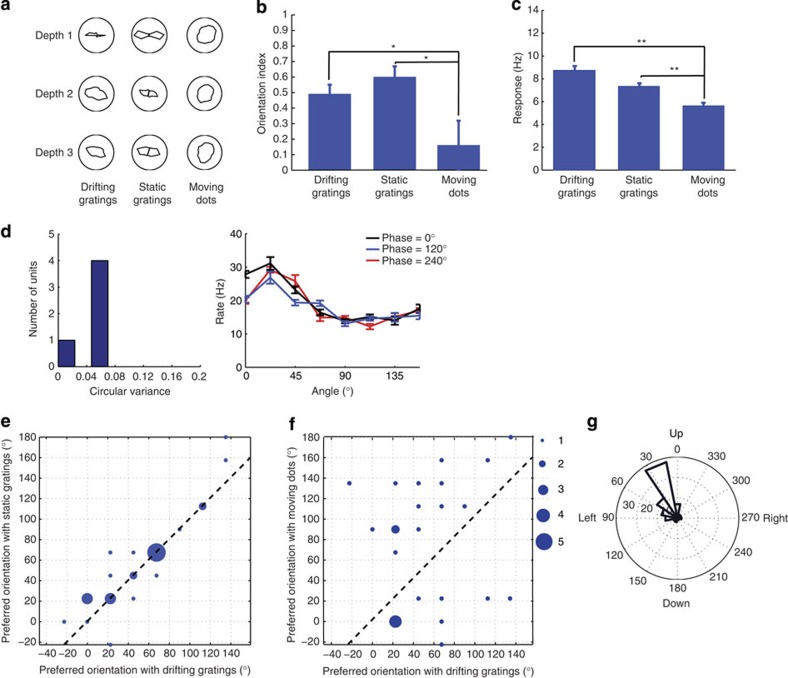
Columns are organized by orientation not by direction. (**a**) Example penetration with tuning for drifting gratings, static gratings and moving dots. For static gratings, the results for any direction and its opposite are the same, and presented only for comparison. (**b**) Orientation selectivity for static and drifting gratings is similar (*P*=0.24, *t*-test, drifting *n*=28 multi-units, static *n*=26, 5 mice) and is lower for moving dots (*P*=0.01 for static, *P*=0.046 for drifting, dots *n*=25). Error bars are mean±s.e.m. (**c**) Mean responses during stimulus presentation are highest for drifting gratings, and similar for static gratings and move dot displays (*P*<0.01 for moving dots compared to drifting and static gratings). (**d**) Histogram of circular variance of preferred orientation of each unit over different phases of static gratings (*n*=5 multi-units, 2 mice; left) and firing rate of an example unit over different angles of the static gratings with three different phases. (**e**) Orientation preference for drifting and static gratings is similar (*n*=26, 5 mice). Size of the dots indicates the number of the units with the same preferred orientation. (**f**) Orientation preference for drifting gratings and moving dots is different (*n*=25 multi-units, 5 mice). (**g**) Direction-selective units (DSI>0.5, left hemisphere) prefer upward motion (*P*<10^−5^, Rayleigh test for circular non-uniformity, *n*=72, 6 mice).

The mouse sSC receives input from direction-selective retinal ganglion cells (DSGCs)^16^,^23^. In the part of the sSC responding to the inferior nasal visual field, we found a dominant preference for upward motion. This could be the direct result of clustering of input from DSGCs preferring upward motion. Apart from this region, however, locations in the sSC responded equally to both directions of the same orientation. Thus, rather than sharing a preference for edge orientation, cells in each column could potentially share a motion axis preference, as recently found in a subset of mouse lateral geniculate nucleus neurons^24^. The functional organization possibly reflects an anatomical clustering of direction-selective retinal ganglion cell axons. The orientation selectivity that we recorded in our units could then result from single sSC cells receiving input from opposing DSGCs. Alternatively, it could result from pooling responses of multiple sSC cells with opposing motion preferences caused by receiving input from one or more DSGCs with a single motion preference. In either case, the units would prefer motion along an axis, rather than the presence of an oriented edge. To distinguish motion axis from edge orientation preference, we performed additional electrophysiological recordings (Fig. 5a). We found that the orientation selectivity for drifting and static gratings was not different (*P*=0.24, *t*-test, drifting *n*=28 multi-units, static *n*=26, Fig. 5b). To confirm that the tuning to static gratings was independent of the spatial phase, we determined the preferred orientation for three different phases (for three recordings the phase was 0°, 120°, 240° and for two it was 0°, 90°, 180°). The low circular variance of the preferred orientation for the different initial phases confirmed that the preferred orientation was independent of spatial phase (*n*=5 multi-units, Fig. 5d). Multi-units preferring a particular orientation of a drifting grating were most often preferring a static grating of the same orientation (Fig. 5a,e). This suggested that cells have a consistent preference for an orientation, independent of the speed of the stimulus. For drifting gratings, motion direction and orientation are directly related and when using drifting gratings it is thus difficult to understand whether cells also would have a matching motion axis preference. For this reason, we presented arrays of moving dots. Although there is no dominant oriented feature in the random arrays, one can still calculate the orientation selectivity index using the motion direction instead of the stimulus orientation. Units preferring a motion axis, that is, motion to diametrically opposite directions, would have a high index. This ‘orientation selectivity’ when measured with moving dots was clearly lower (*P*<0.05, *t*-test, gratings *n*=28 multi-units, dots *n*=25, Fig. 5b) than when measured with gratings, whereas the cells showed robust mean responses to all stimulus types (Fig. 5c). Also, the preferred orientation, defined as the orthogonal orientation to the preferred direction, for moving dots was generally different to the preferred grating orientation (Fig. 5a,f). We conclude that the selectivity is for orientation and not for motion axis.

In our recordings across animals, there was a clear bias for upward motion in direction-selective cells (*P*<10^−5^, Rayleigh test for circular non-uniformity, *n*=72 multi-units, Fig. 5g). This was as previously reported in one study^5^ and attributed to retinal input^23^, but not in another^15^. Our results show that the presence of this bias in the data set depends on how many cells with a preference for the inferior nasal visual field were recorded. Outside this field, neurons display an orientation preference rather than a direction preference. In the nasal visual field, we observed a preference for horizontal gratings moving up or down, for the temporal visual field this is for vertical gratings moving sideways to the front or to the back. In general, cells have a strong concentric bias and prefer gratings that are orthogonal to the radial line from their receptive field position to the centre of vision. One caveat is that our recordings were done with a large screen positioned directly in front of the mouse. The gratings had a homogeneous physical size across the screen. The perceived spatial frequency in the periphery was thus different from the spatial frequency in the centre, and the spatial frequency in the receptive field of a neuron for a grating parallel to the concentric circle around the nose through the receptive field would be higher than for a grating parallel to the radial line from the nose to the receptive field (Supplementary Fig. 10a,b). A calculation of the size of this effect (see Supplementary Note 1 and Supplementary Fig. 10c,d), however, suggests that this distortion is unlikely to be the source of the bias. Furthermore, for a number of electrophysiological recordings, we have first measured the orientation preference with the screen in our standard configuration, and then angled the screen, both horizontally and vertically, to be close to orthogonal to the radial line from the nose to the receptive field location. This had no little influence on the orientation preference (*t*-test, *P*=0.30, 14 multi-units, 3 mice, Supplementary Fig. 10e).

## Discussion

We found that in the mouse sSC there is a strong bias for a neuron to prefer gratings, which are oriented parallel to the concentric circle around the centre of the visual field through the receptive field of the neuron. Surprisingly, many combinations of retinotopic position and preferred orientation were not encountered, suggesting a different layout and origin than the orientation maps in V1 of carnivores and primates^24^,^25^,^26^,^27^,^28^. Neither we nor any of the previous sSC imaging studies^29^,^30^ left much space for the possible existence of secondary retinotopic maps in the sSC that are sufficient in size to cover all other angles. Therefore, it appears that uniform coverage for all angles and positions is absent in the sSC. Concurrently with our finding, a study imaging the medial-caudal part of the SC came to the same conclusion^31^. They showed that there is a columnar organization of orientation preference, but for the lateral visual field to which the area they imaged responded, there is no consistent preference for concentric orientations, differently from the more central visual field where we did our recordings. The absence of uniform coverage of angles and positions found in these two studies suggests that the mouse sSC is unsuited for translation-invariant object recognition. Rather than coding for all orientations and positions, the relationship between preferred orientation and direction, and retinotopy resembles a combination of an expanding and receding optic flow map, where for each retinotopic position the preferred orientation is one for which the corresponding orthogonal movements are straight towards and away from the nose. The computation of such a map may help in gaze orienting and smooth pursuit^32^, roles ascribed to the SC^33^. The neural tunings would help to gauge the optic flow caused by movement of the eyes, head or body. Interestingly, a similar, but considerably weaker bias for centrifugal or centripetal motion is present in the primate frontal eye field^34^, a cortical area involved in eye movement control.

Apart from a role in smooth pursuit and gaze orienting, the observed layout of the sSC might also subserve avoidance and escape behaviour, as the rodent SC plays a role in such behaviour^10^. The specialization for orientation for each part of the visual field may help to optimally detect predators or other animals that are approaching from the front or receding from that direction. In this way, the concentric orientation bias could be a heuristic for computing the need to initiate avoidance and escape manoeuvers. Such dependence of escape manoeuvers on visual field location has been observed in rats, which respond with immediate evasive action to drifting stimuli overhead, while they ignore gratings shown at nose level^35^.

An open question is how much of the observed organization of the mouse SC is shared with other species. Some vertical grouping of direction selectivity has been described 40 years ago in the ground squirrel, a diurnal rodent^36^. This suggests that rather than mouse specific, the organization of orientation may generalize across rodents, although there are no reports of such organization in the rat to date. The organization of orientation that we report here for the mouse, however, is unlikely to be present in the cat or the macaque. There has been extensive exploration of the SC in these species, in particular looking at the integration of different modalities across layers^6^,^37^. Therefore, it is highly unlikely that a similar organization of the SC in these species has remained undiscovered. In cat retina^38^ and dorsal lateral geniculate nucleus^39^, and in monkey^40^ and human^41^ visual cortex, both an overrepresentation of preferences for concentric orientations and centrifugal and centripetal motion are reported, as well as a bias for radial orientations, that is, gratings parallel to the radial line connecting the centre of vision to the receptive field location. These biases have not yet been reported for the mouse.

There are a number of other differences between rodents and the other mammalian species mentioned above. Most conspicuous are the large fraction of retinal axons that project to the sSC, the large tectal volume compared with cerebral cortex size, and the strong direction and orientation selectivity in the rodent superficial lamina of the SC. These differences suggest a more important role for the SC in vision in the rodent, although it is not clear if this is causally related to its organization of orientation.

Another species difference is the dependence of tectal response on the cortex. We found no reduction of orientation tuning in the sSC resulting from cortical silencing. This is consistent with other work in the mouse, where no difference in orientation selectivity was detected upon ablation of the visual cortex, and even a small increase in direction sensitivity was apparent^15^. In the ground squirrel, the responses in the sSC were also not affected by cortical removal, and cells remained direction selective^42^. In primate, where there is relatively little direction selectivity and visual feature selectivity, tuning was also not much affected^43^. This is in contrast to the effects in the cat, where cortical cooling or ablation removed direction selectivity almost completely^44^, although topical silencing of visual cortex did not^45^. This difference points to a different origin of the orientation and direction selectivity of the sSC in carnivores and rodents.

The situation in the mouse SC resembles to some extent the situation in a species without a neocortex. In the tectum of zebrafish larvae, direction- and orientation-selective input from the retina is clustered by preference and shows strong retinotopic biases^46^. The orientation and direction selectivity in the tectal cells themselves match this input to a large degree^47^, but at least at the larval stage there is no simple mapping between retinotopy and preferred orientation^48^.

In the zebrafish, direction- and orientation-selectivity are both inherited from the retina as well as generated locally in the tectum^47^. It is not yet known whether the orientation selectivity in the mouse SC is inherited from the retina or computed locally. Orientation-selective retinal ganglion cells are present in the mouse retina^49^. They have not yet been genetically isolated or filled with tracer to see if they project to the sSC, but given the large number of retinotectal projections it is likely that they do. The orientation-tuning in the sSC could also be achieved by grouping of inputs with opposing motion preferences from DSGCs. There are several subtypes of DSGCs that direct to the sSC and which have specific motion preferences and genetic identity^23^,^50^. Clustering of their terminals in the sSC could explain the orientation and direction anisotropies in the sSC. It is known that the projections of a number of retinal ganglion cells, such as the tOFF-alphaRGCs^16^ and the TRHR On-Off pDSGCs^17^ terminate in patches in the sSC. Moreover, existing anisotropies in the retinal distributions of ganglion cell subtypes^51^ and the clustering of retinal input have been used to explain part of the anisotropy in the fraction of direction- and orientation-selective cells^52^ and in preferred direction^24^,^53^ in the mouse dLGN^24^,^49^,^52^. Based on the axonal projections to the sSC, at least four parallel functional maps in different sublayers of the sSC were hypothesized to exist^18^. Imaging of activity in the sSC in mice with genetically labelled retinal ganglion cells will reveal how the maps of visual space are laid out in the SC and match with the observed functional map of collicular responses.

In summary, we have found that in the rodent sSC orientation and direction preference are tightly coupled to receptive field location, and that there is no uniform coverage of all preferred angles over the full visual field. We expect that this link in visual position and orientation and the lack of coverage of all angles and positions will have behavioural consequences, but these remain to be discovered.

## Methods

### Animals

C57BL/6JOlaHsd (Harlan) and C57BL/6J (Charles River) male mice of 6–12 weeks old were used for the experiments. Mice were housed in 12 h/12 h dark/light cycle. All experiments were approved by the Institutional Animal Care and Use Committee of the Royal Netherlands Academy of Arts and Sciences. All animals were used for either electrophysiology or imaging.

### Electrophysiology surgery

Mice were anaesthetized by an intraperitoneal injection of 1.2 g urethane per kg body weight, supplemented by a subcutaneous injection of 8 mg chlorprothixene per kg body weight^54^. We injected atropine sulfate (0.1 mg per kg) and dexamethasone (4 mg per kg) subcutaneously to reduce mucous secretions and to prevent cortical oedema, respectively^55^. Additional doses of urethane were injected when a response to toe-pinch was observed. Mice were head fixed by ear and bite bars. Temperature was measured with a rectal probe and maintained by a feedback-controlled heating pad set to 36.5°. A total of 14 mice were used to produce Fig. 1f–h. This included the animals used for Fig. 5. The three animals used for Fig. 4h–j were not included in the 14 animals used for Fig. 1.

### Electrophysiological recording

Laminar silicon electrodes (A1 × 16-5mm-50-177-A16, 16 channels spaced 50 μm apart, Neuronexus) and tungsten in glass microelectrodes (Alpha-Omega) were used for extracellular recordings. For recordings perpendicular to the tectal surface, electrodes (37 tungsten microelectrode and 20 silicon probe penetrations) were inserted through a craniotomy 200–1,200 μm lateral and 100–900 μm anterior and 1,000–1,800 μm down from lambda. There was considerably less visual response 300 μm below the top of the sSC. This means usually around 7–8 channels of the laminar probe were within the sSC. We have no knowledge of the exact depth of our recording sites relative to the sublamina (zonal, upper and lower superficial grey and optic layers) of the sSC. Horizontal recordings were made by inserting tungsten micro-electrodes along the lateral-medial axis through a craniotomy 400–900 μm anterior to lambda. In these penetrations, orientation preference was determined at 30–50 μm intervals with 22.5° resolution. Recordings for directions vs orientation tests were done with tungsten microelectrodes. Laminar probe signals were amplified and filtered at 500 Hz–10 kHz and digitized at 24 kHz using a Tucker-Davis Technologies RX5 pentusa. Tungsten microelectrode recordings were amplified (MCP Plus, Alpha Omega), filtered at 300 Hz–10 kHz and digitized at 33 kHz (1401, Cambridge Electronics). Signals were thresholded at 3x standard deviation to isolate spikes, and spikes were sorted by custom-written Matlab (Mathworks) scripts, but single and multi-units were pulled together for this publication to increase the number of orientation measurements on a single penetration. Minimum evoked visual response for a unit to be included was 2 Hz.

### Visual stimulation for electrophysiology

Stimuli were projected by a gamma-corrected PLUS U2-X1130 DLP projector onto a backprojection screen (Macada Innovision), positioned 15 cm in front of the mouse. The full-screen, square wave, grating stimuli produced using Matlab Psychophysics Toolbox 3 (ref. 54) were 0.05 cycles per degree, 90% contrast and covered a 75 × 57 cm^2^ area. The spatial frequencies of the stimuli were computed for the shortest distance of the screen to the animal, and the widths of the bars was constant across the screen. Drift speed was 2 Hz. For the static grating stimuli of Fig. 5, we used a single phase except noted otherwise (Fig. 5d). The motion stimuli used for Fig. 5 consisted of a display of square white dots (~2°) randomly placed to cover approximately 30% of the screen, all moving in the same direction at ~30° s^−1^. Interstimulus time was 1 s. Background luminance was 10 cd m^−^^2^. To determine receptive field location, we presented a 5-min movie (five frames per second) of small white squares (5°) in random positions with a black background (ratio of white to black area: 1/30).

### Optogenetics and drug delivery

For silencing the visual cortex by optogenetics, six mice were anaesthetized with isoflurane (5% induction, 1.5–2.5% maintenance) and one to three small craniotomies were made above the visual cortex in each of these. Using a Drummond Nanoject volume injector at each of five depths of visual cortex, 18–54 nl was injected of a solution of adeno-associated virus with an ArchT^56^ vector behind a CaMKII promoter to drive expression in excitatory neurons (AAV1.CAMKII.ArchT.GFP.WPRE.SV40, 6.4 × 10^−2^ GC per ml, University of Pennsylvania Vector Core). The scalp was resutured and the vector was allowed to express for several weeks before acute electrophysiology. An orange (617 nm) fibre-coupled LED (Thorlabs) was used to activate the ArchT pump. Trials with light on were intermingled with trials with light off. In light on trials, the light was on 1 s before stimulus onset until stimulus offset. To have a broader silencing of visual cortex, we injected 1 μl of 40 mM muscimol (an agonist of GABA_A_ receptors) in V1 (2.9 mm lateral and 0.4 mm anterior to Lambda) by a Drummond Nanoject volume injector (with volume rate of 2.3 nl per second). This was done while laminar probes were present in the sSC and V1.

### Imaging surgery

To prepare the mice for calcium imaging, a two-stage surgery was performed. First, using the same procedure described above in the optogenetics section, we injected a virus for expression of the calcium indicator GCaMP6s (AAV1.Syn.GCaMP6s.WPRE.SV40, 1.9 × 10^−3^ GC per ml, University of Pennsylvania Vector Core) about 100 nl in three to five loci in the right sSC in four different depths. The surgery was performed under 1.5–2.5% isoflurane anaesthesia. After the injections, we resutured the scalp and the animals recovered and were kept in the cages for 3–5 weeks to get sufficient expression of GCaMP6s. A second surgery was necessary directly before the imaging session to get access to the sSC and to implant a head ring and cranial window for calcium imaging. The mice were anaesthetized by an intraperitoneal injection of 1.2 g urethane per kg body weight, supplemented by a subcutaneous injection of 8 mg chlorprothixene per kg body weight. We injected atropine sulfate (0.1 mg per kg) and dexamethasone (4 mg per kg) subcutaneously to reduce mucous secretions and to prevent cortical oedema, respectively. Mice were head fixed by ear and bite bars. Temperature was measured with a rectal probe and maintained by a feedback-controlled heating pad set to 36.5°. A metal ring of 15 mm outer diameter and 9 mm inner diameter was fixed on top of the skull with dental cement and glue for the purpose of head-fixation during imaging. After making a craniotomy of 4–5 mm^2^, we very carefully lifted and sucked away the cortex (around 1–2.5 mm^2^) above the sSC. We used artificial cerebrospinal fluid (ACSF) to rinse any blood off the sSC surface. We filled the craniotomy by a solution of 1% agarose in ACSF and cemented a round glass (with diameter of 5 mm) on the skull on top of the craniotomy. After surgery, the mouse was kept anaesthetized and quickly transferred to the wide-field imaging setup. Surgery was started on 21 animals, but only 7 animals had expression, an undamaged sSC and survived to the stage of imaging.

### Wide-field imaging

To visualize the retinotopic map and the horizontal structure of the orientation domains, we imaged the changes of calcium levels in response to visual stimuli using GCaMP6s. We used a blue LED with a blue filter (482±25 nm) for excitation and measured the calcium activity through a 495-nm dichroic mirror and a green band-pass emission filter (525±45 nm). Images were collected using a CCD camera (Teli, 11.04 μm per pixel) at 1.8 Hz acquisition rate through × 1 macroscope lense assembly using VDAQ acquisition system (Optical Imaging Inc, http://www.opt-imaging.com). A large (42 inch) gamma-corrected M4210LG display screen (LG) was placed 29.5 cm in front of the mouse. We first obtained a coarse retinotopic map to see for which part of the visual field we were recording responses, by showing at the four quadrants of the monitor, five or more repetitions of 6 s long, 0.05 c.p.d. square-wave gratings drifting at 40° s^−1^, changing direction every 0.6 s, as used previously^57^. Based on this coarse map, we sometimes repositioned the monitor to cover receptive fields of the responsive area, and started a finer retinotopic mapping. To get the horizontal structure of orientation preference, we showed full-screen grating drifting in eight different directions. The spatial frequency, contrast and drift speed were 0.05 cycles per degree, 90% and 2 Hz, respectively, except for the experiments shown in Supplementary Fig. 8, where we also used 0.1 and 0.16 c.p.d. For retinotopic maps, at least five repetitions of all patches were shown, and for the orientation imaging at least 20. Stimulus duration was 3 s and we used a minimum interstimulus interval of 9 s.

### Data analysis

Analysis was done using custom Matlab scripts, a fork from code written by Steve Van Hooser, and available online at https://github.com/heimel/InVivoTools. Circular mean of the preferred orientation of a penetration (that is, a single probe insertion or tungsten track) was computed as half the angle of ∑_*p*_exp(2i*ϕ*_*p*_), where *ϕ*_*p*_ is the preferred angle of recording position *p* for the tungsten microelectrode or channel for the linear probe. The circular variance for a single penetration or track was computed as 1− |∑_*p*_exp(2i*ϕ*_*p*_)|/*n*, where *n* is the number of channels or positions recorded on the penetration. We then calculated the median circular variance for all penetrations. Next, 10,000 × times all orientation preferences of all positions on all penetrations were randomly redistributed over all positions on all penetrations. The resulting distribution is shown in Fig. 1g. The histogram of the median circular variance for all penetrations for each reshuffling is shown in Fig. 1h.

Orientation index was defined as *OI*=(*R*_Pref_ −*R*_Ortho_)/(*R*_Pref_+*R*_Ortho_), where *R*_Pref_ is the average neuron’s response to the preferred orientation (that is, two opposing direction for drifting gratings) and *R*_Ortho_ is the average neuron’s response to the orientation orthogonal to the preferred orientation. Cells were called direction selective if their direction selectivity index (DSI) was larger than 0.5, where DSI=✓((∑_*ϕ*_*R*(*ϕ*) sin(*ϕ*))^2^+(∑_*ϕ*_*R*(*ϕ*) cos(*ϕ*))^2^) / ∑_*ϕ*_*R*(*ϕ*), where *ϕ* is the angle of the stimulus, *R*(*ϕ*) is the neuron’s response to it, and ∑_*ϕ*_ denotes the summation over all the represented angles. For Supplementary Fig. 10e, the preferred orientations were computed from Von Mises function, exp(cos(2(*ϕ*-*ϕ*_preferred_))−1), fits to the orientation tuning curves.

For the macroscopy, the responses were the averages over all repetitions of the mean change in fluorescence from 0.3 s after stimulus onset until 2 s after stimulus offset divided by the mean 3 s baseline fluorescence before the stimulus, that is, response for repetition *i* of stimulus *s*, which lasts from *t*_onset_ to *t*_offset_, was (*R*_s_-*R*_0_)/*R*_0_, where *R*_s_ is the average image intensity over the region of interest from *t*_onset_+0.3 s to *t*_offset_+2 s and *R*_0_ is the average image intensity over the region of interest from *t*_onset_−3 s to *t*_onset_+0.3 s. This response was divided by 1 plus the same response in a visually not responsive reference region, to correct for slow changes in fluorescence unrelated to neuronal activity. Maps were made by spatially filtering by a convolution with a 2D gaussian with sigma of 33 μm (3 pixels). For the retinotopic maps in Figs 3b and 4a, every pixel was coloured with a hue assigned to the patch on the monitor to which it was most responsive. The saturation of the pixel was linearly scaled according to the level of the response, with the maximum saturation matching the maximum response. For the orientation maps shown in Figs 3d and 4e and Supplementary Fig. 7, the mean response for each pixel to each of the eight directions were computed as described above. Next, for each pixel *p*, we computed *R*(*ϕ*,*p*) as the maximum response of each orientation *ϕ* to the two corresponding directions. Finally, we computed the angle of ∑_*θ*_ R(*ϕ*,*p*) exp(2i*ϕ*). This angle was used to determine the hue of each pixel. The saturation was scaled according to the level of the mean response of all stimuli. For the significance map in Fig. 3d, the *P*-value of the ANOVA across orientations for all repetitions was computed and thresholded. The single-condition maps in Fig. 3c show the mean response during the selected directions from which the mean response over all directions is subtracted. Figure 3e was constructed by taking the ROI for each of the retinotopic patches in Fig. 3b and computing the average tuning curve for all pixels in each region. The preferred orientation was then drawn in the corresponding patch on the monitor. The concentric angle maps in Fig. 4 and Supplementary Fig. 7 were created by automatically detecting the centre of response for each monitor patch by fitting the response with a 2D Gaussian, where we first subtracted 0.1% of the response to obtain better fits. For the fitting, we used a matlab function adapted from http://jilawww.colorado.edu/bec/BEC_for_everyone. Patches with a fitted response of at least 0.4% were included for calculation of retinotopic map. With aid of the Matlab function, TriScatteredInterp smooth maps of the corresponding receptive field locations on the monitor were constructed. The cut-out of these maps from the original full-image was done automatically by TriScatteredInterp, which gives the results of a 2D interpolation. Occasionally, the Gaussian fits would produce a fold in the map, but the interpolation function removed these folds. For each position on the sSC, the corresponding position on monitor was computed in *x,y,z* coordinates with respect to the nose (with *z* the axis of the body of the animal, *x* to the right of the animal and *y* above the animal). From these *x,y,z* coordinates, we computed the azimuth and elevation through a standard cartesian to spherical coordinate transform, where azimuth=arctan(*x*/*z*), elevation=arctan(*y*/sqrt(*x*^2^+*z*^2^)) to produce Fig. 4b,c and Supplementary Fig. 7. The concentric angle map in Fig. 4d and Supplementary Fig. 7 was calculated by transforming the original cartesian *x,y,z* coordinates to cylindrical coordinates by as (*π*/2− cart2pol(*x*,*y*))/π*180, where cart2pol is a Matlab function using the four quadrant arctangent of *x* and *y*. For some animals, we had not recorded the exact pitch and roll of the head. For these animals, we chose the values that best fitted the concentric map hypothesis. The results, however, are fairly independent of this, because when we replace all rolls by the mean roll for all experiments, the circular correlation coefficient of measured preferred orientation and computed concentric orientation only drops by 4% and thus remains high. Also if we change the pitch of all imaged animals by 10° up or down, the circular correlation coefficient varies by maximally 20%. An optimal fit was achieved by further smoothing the radial angle map by convolving with a 2D Gaussian with a sigma of 132 μm. This spatial filtering simulates the scatter of retinotopic position and light scatter in the images. We chose its specific size because it made the computed concentric orientation map and measured orientation map of the animal shown in Fig. 4d,e visually look most alike, but it is likely to overestimate the light scatter and rf position scatter. The quantification, however, is fairly independent of the spatial filter as the circular cross correlation only drops by 6% if no smoothing is used. For each of the pixels in the cut-out, we could plot the calculated radial angle and measured orientation (Fig. 5f). For quantification of similarity in Supplementary Fig. 8, a minimum mean response of 0.5% was required for each condition.

Two-sided Student’s *t*-tests were used to compare orientation selectivity for the static and drifting gratings, and the moving dots. Wilcoxon signed rank sum test was used as the non-parametric paired test to compare median of penetrations when cortex was optogenetically silenced versus when it was not, because the distribution of medians was bound from below and clearly not normal. The Mann–Whitney *U*-test was used for unpaired comparisons of orientation index and response levels, which were not normally distributed. An ANOVA was used if the data from all groups passed the Shapiro-Wilk normality test.

## Author contributions

M.A. initiated the electrophysiological experiments and performed all surgeries and analyses. J.A.H. and M.A. devised the experiments and analyses, and performed the imaging. J.A.H. wrote the manuscript.

## Additional information

**How to cite this article:** Ahmadlou, M. *et al.* Preference for concentric orientations in the mouse superior colliculus. *Nat. Commun.* 6:6773 doi: 10.1038/ncomms7773 (2015).

## Supplementary Material

Supplementary Information Supplementary Figures 1-10, Supplementary Note 1 and Supplementary References
