# Supplementary material for: Preference for concentric orientations in the mouse superior colliculus
Source: Nat Commun. 2015 Apr 2;6:6773. doi: 10.1038/ncomms7773 (PMC4396361; doi:10.1038/ncomms7773)
Supplement: Supplementary Information — Supplementary Figures 1-10, Supplementary Note 1 and Supplementary References [file ncomms7773-s1.pdf]

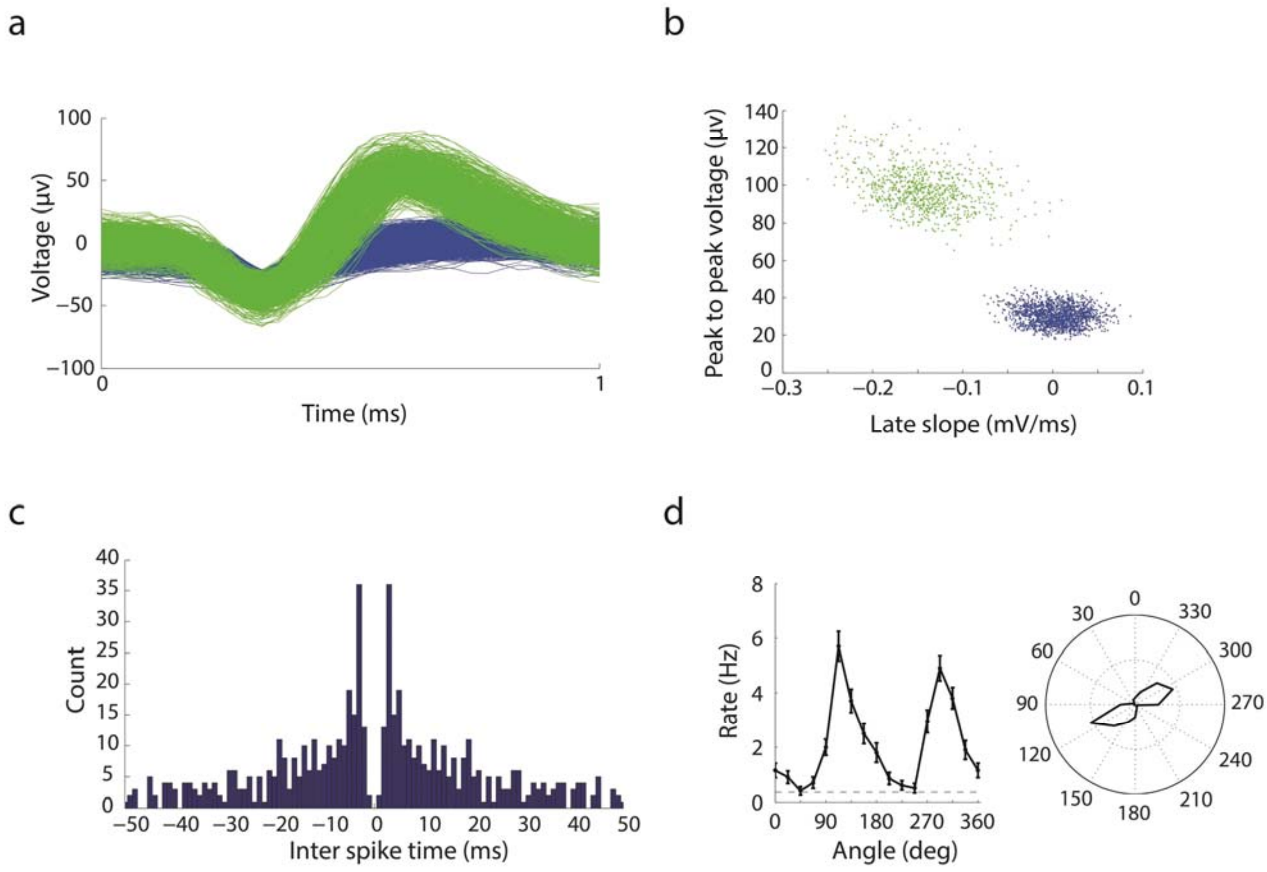

Supplementary Figure 1 Single-unit selection and orientation tuning. **(a)** An example of single-unit (green) and multi-unit (blue) wave forms recorded during presentation of drifting gratings. **(b)** The single-unit and multi-unit waveforms show two separate clusters in space of peak to peak voltage versus late slope. **(c)** Histogram of inter spike time intervals of the single-unit. **(d)** Firing rate (left) and orientation tuning (right) of the single-unit.

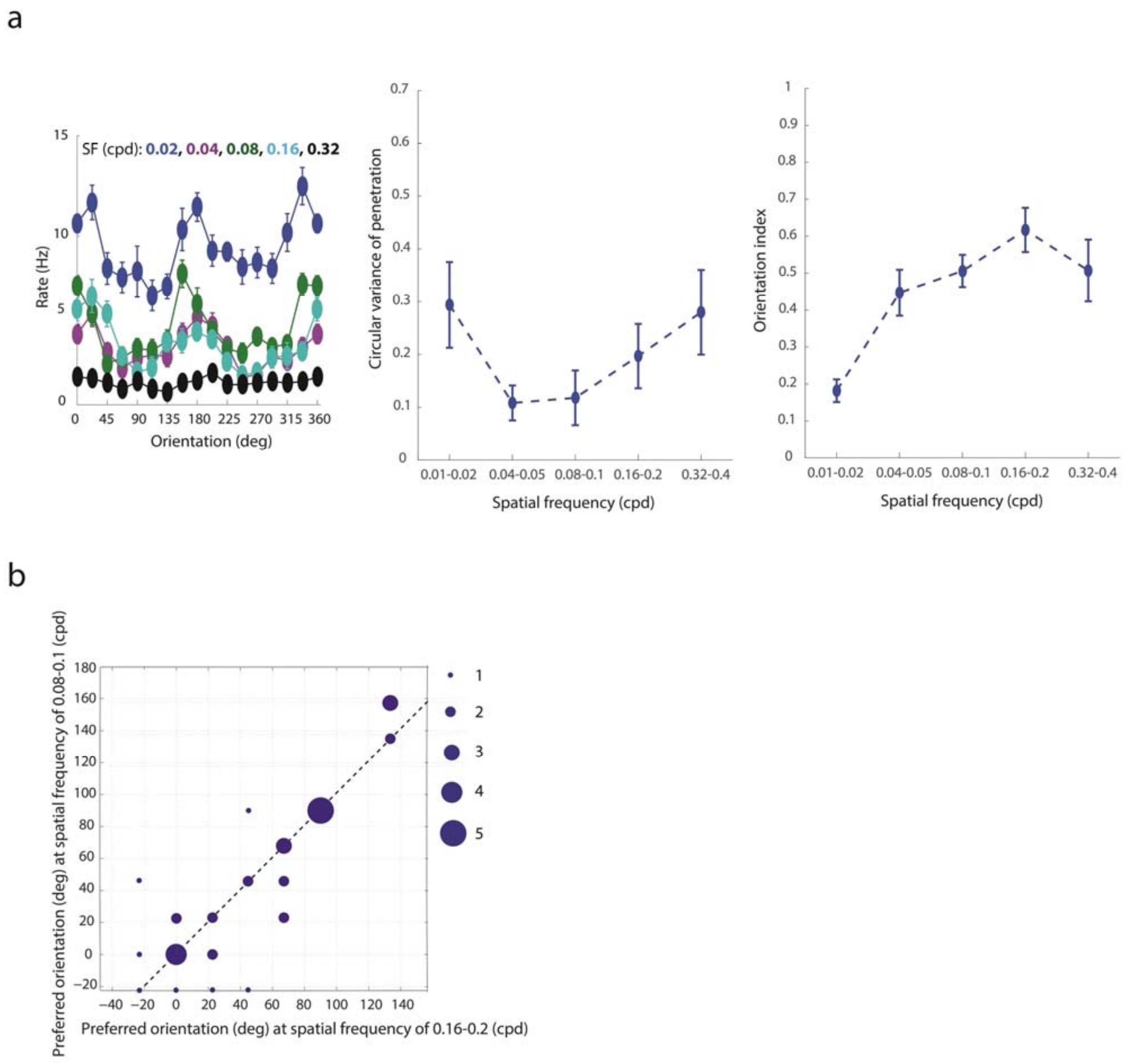

Supplementary Figure 2 Orientation columns in sSC can be observed over a range of spatial frequencies (SF). **(a)** Left panel shows responses of one example unit to different orientations when SF varies (0.02, 0.04, 0.08, 0.16, and 0.32 cpd). Note that square wave gratings were used. Error bars are mean +/- SEM. Middle and right panels show for different SFs the circular variance (CV) of the orientation preferences of units for 10 vertical penetrations (i.e. 1 CV value per penetration, 6 mice) and the mean orientation index (OI) of the units on these penetrations. For 7 vertical penetrations (4 mice) the SF set is [0.02, 0.04, 0.08, 0.16, and 0.32 cpd] as well as the standard SF of 0.05 cpd and for 3 vertical penetrations (2 mice) the SF set is [0.01, 0.1, 0.2, and 0.4 cpd]. **(b)** Orientation preference shows little change when SF is changed from 0.08 to 1.6 cpd, or from 0.1 to 0.2 cpd (n=36 units, 6 mice). Size of the dots indicates the number of the units for each specific pair of preferred orientations.

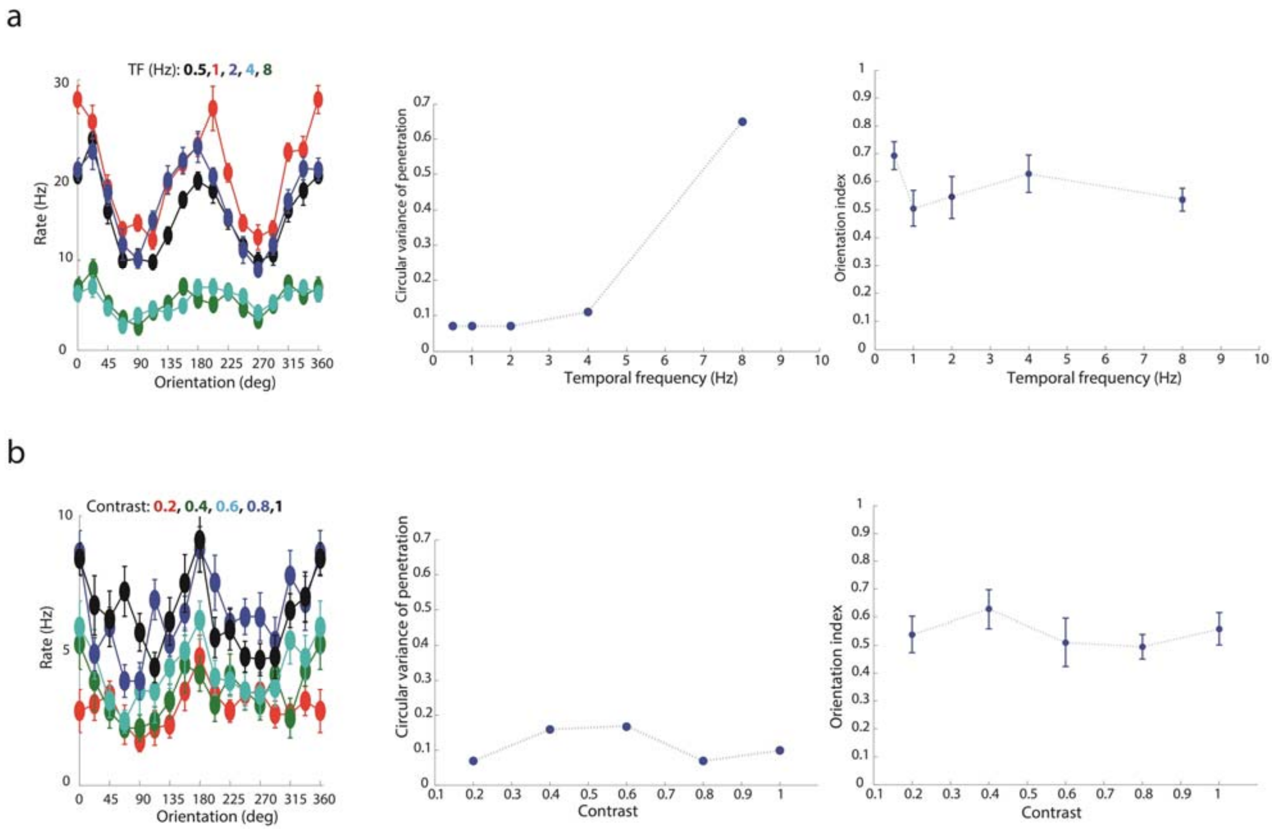

Supplementary Figure 3 Orientation columns in the sSC can be observed over a large range of temporal frequencies (TF) and contrasts. **(a)** Left panel shows response of one example unit to different orientations when TF varies (0.5,1, 2, 4, and 8 Hz). Error bars are mean +/- SEM. Middle and right panels show circular variance (CV) of one vertical penetration and the orientation index (OI) of units on this penetration (1 mouse) over different TFs. **(b)** Left panel shows response of one example unit to different orientations when contrast varies (20, 40, 60, 80 and 100 %). Error bars are mean +/- SEM. Middle and right panels show CV of one vertical penetration and the OI of units on this penetration (one mouse) over different contrasts.

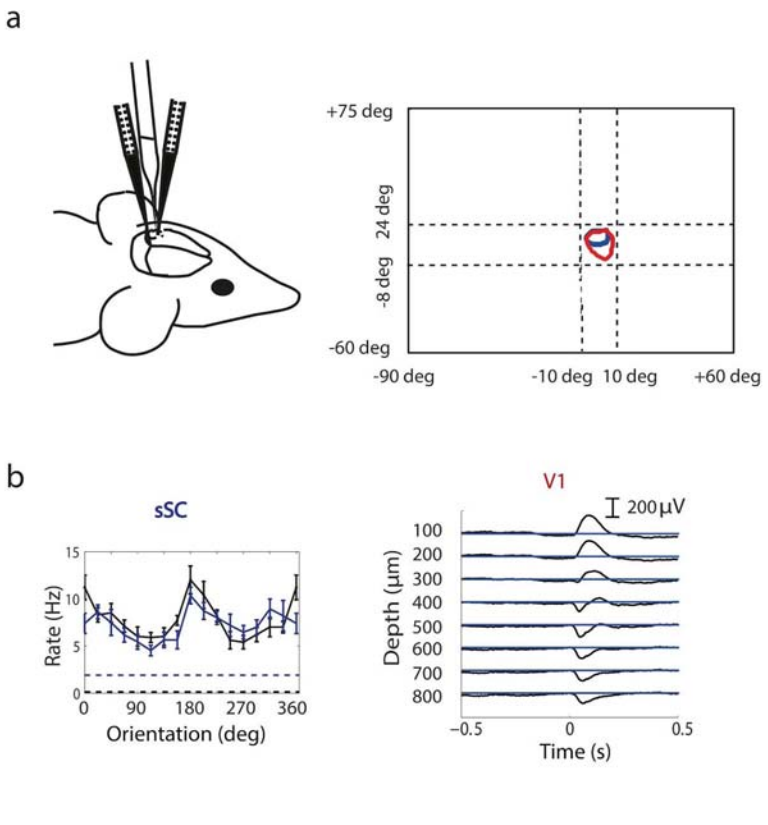

Supplementary Figure 4 Silencing of visual cortex with muscimol does not affect vertical grouping of orientation. **(a)** Two recording probes are placed in matching retinotopic positions in V1 and the sSC, while an injection pipette was placed above V1. The right panel shows the ON-response fields for one V1 site (red) and sSC site (blue) during a simultaneous recording (1 mouse). **(b)** Responses of multi-units in the sSC (left; error bars are mean +/- SEM.) and visual evoked potential in all layers of V1 (right) before (black) and 5 minutes after (blue) injection of muscimol in V1. It shows that V1 is silenced, while sSC remains active (1 mouse). **(c)** Example tuning curves from 7 depths in the sSC, separated by 50 µm, before (left) and after (right) silencing V1.

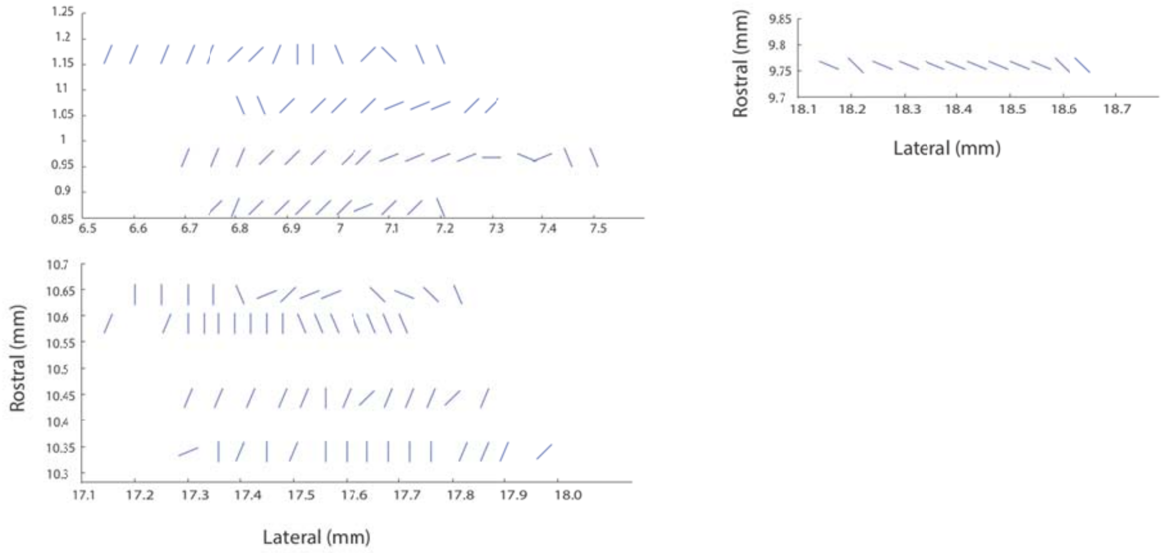

Supplementary Figure 5 Examples of orientation preferences (measured at 22.5 deg resolution) encountered during horizontal penetrations (123 units; 2 mice). Offsets are arbitrary.

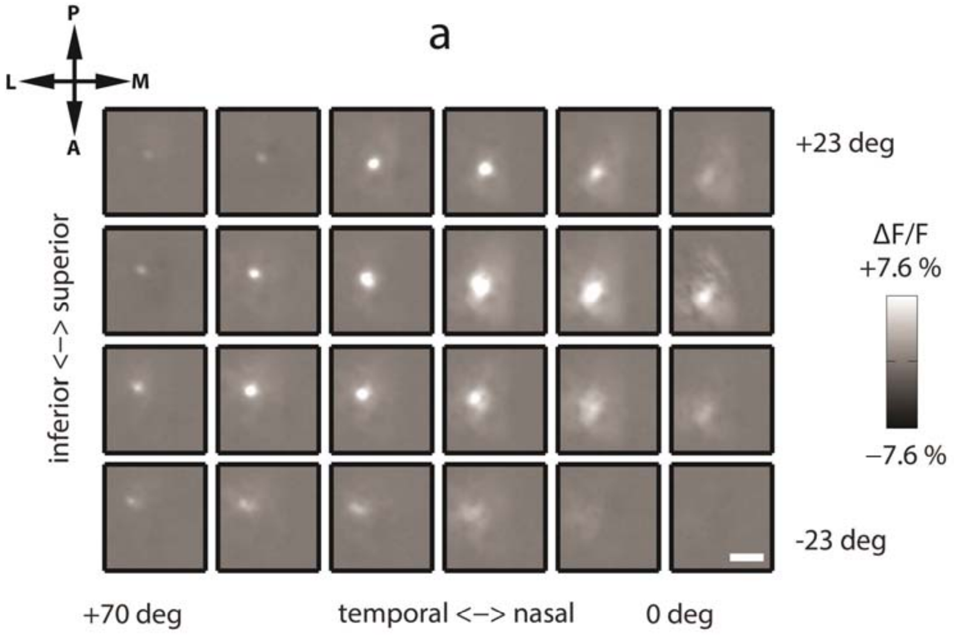

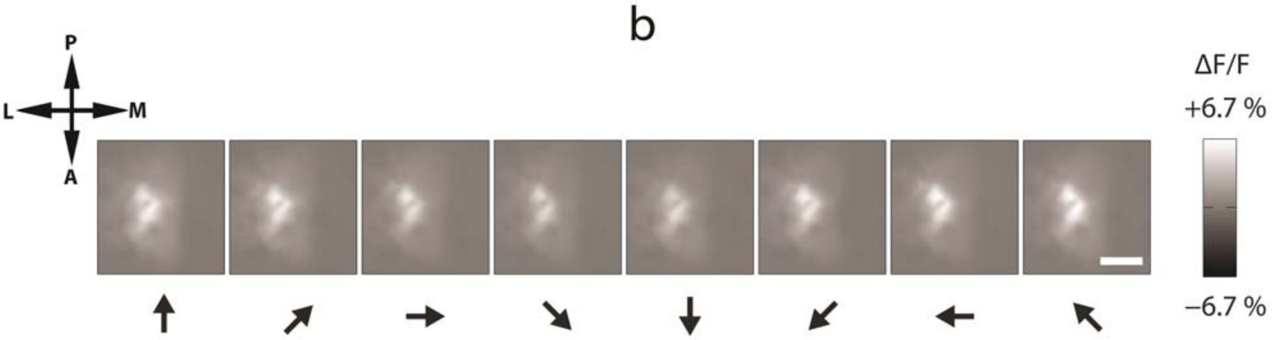

Supplementary Figure 6 Single condition responses of macroscopic calcium imaging. **(a)** Single condition responses to retinotopically restricted stimuli, used to compute Figure 4b. Intensity bar shows the *ΔF/F* in percentage and scale bar is 1 mm. **(b)** Single condition responses of mouse of Figure 4 to orientation stimuli. Intensity bar shows the *ΔF/F* in percentage and scale bar is 1 mm.

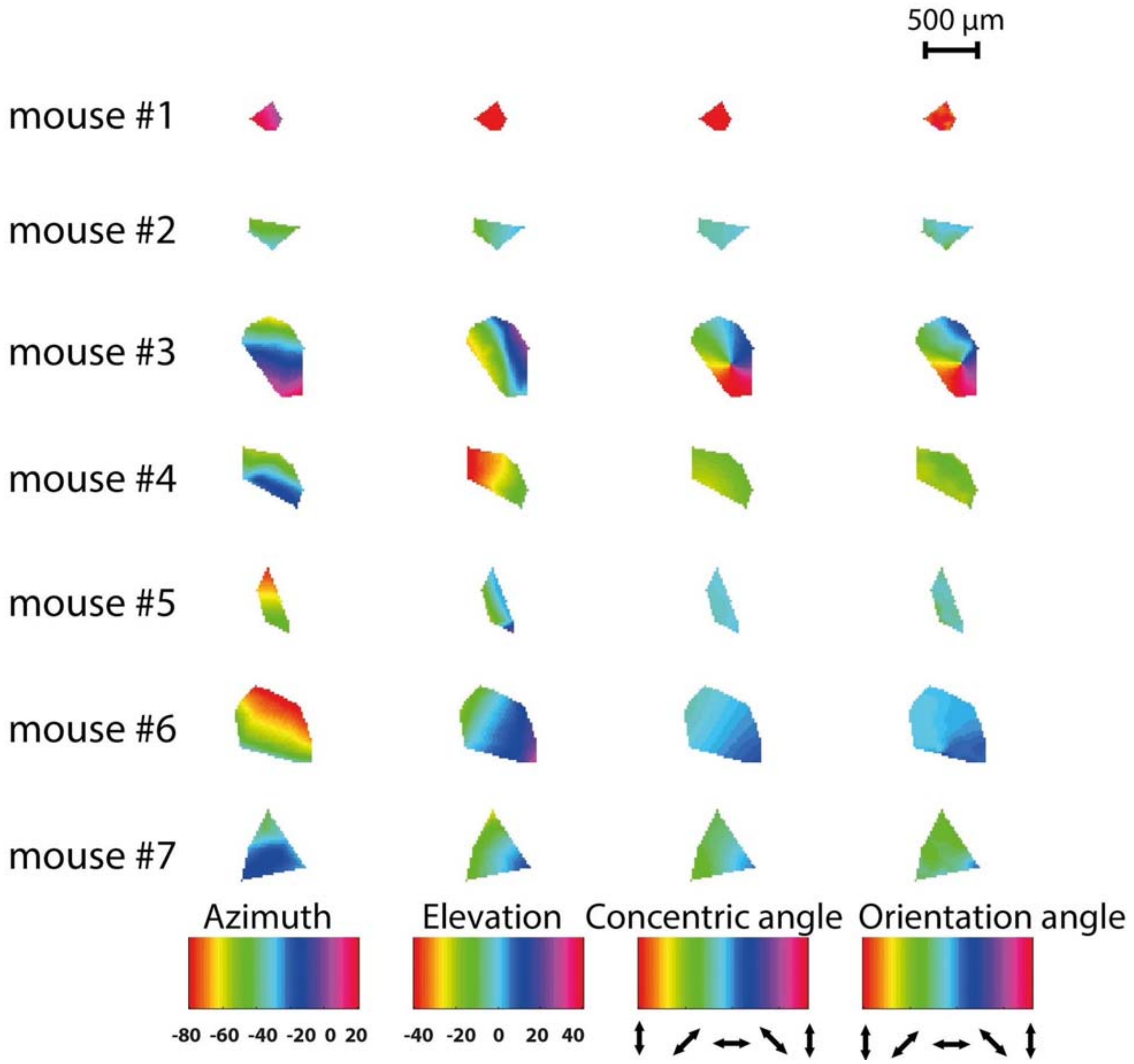

Supplementary Figure 7 Partial orientation maps of 7 mice. From left to right, retinotopic map in azimuth axis and elevation axis, the concentric angle map reconstructed based on the retinotopic map in azimuth-elevation coordination, and the preferred orientation map.

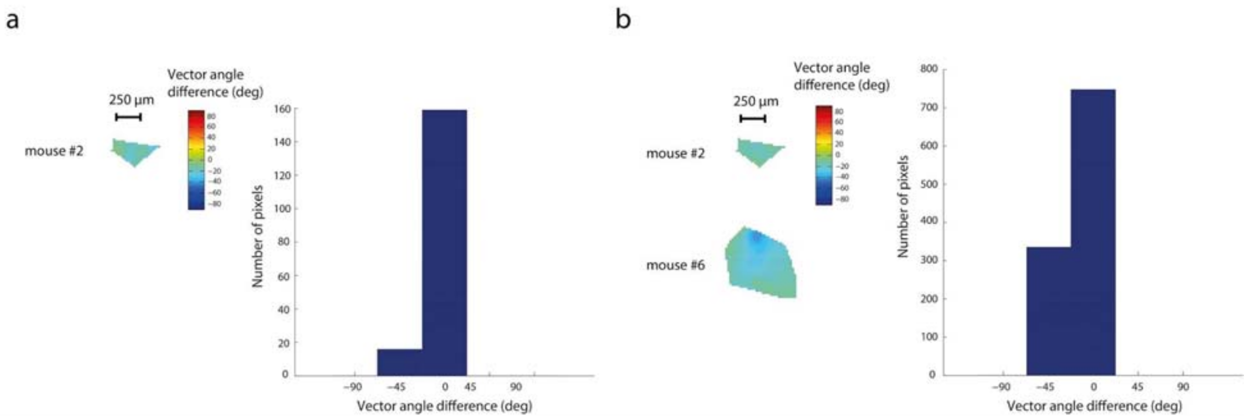

Supplementary Figure 8 Robustness of vector angle map to changes in phase or spatial frequency. **(a)** Left: difference of vector angle of each pixel in one mouse (mouse #2) in response to drifting gratings with two different phases (180 deg apart). Right: Histogram of vector angle difference of the maps shown in the left panel. **(b)** Left: difference of vector angle of each pixel in 2 individual mice in response to drifting gratings with two different spatial frequencies. The spatial frequencies for mouse #2 are 0.05 and 0.16 cpd and for mouse #6 are 0.05 and 0.10 cpd. Right: Histogram of vector angle difference of both maps, together, shown in the left panel.

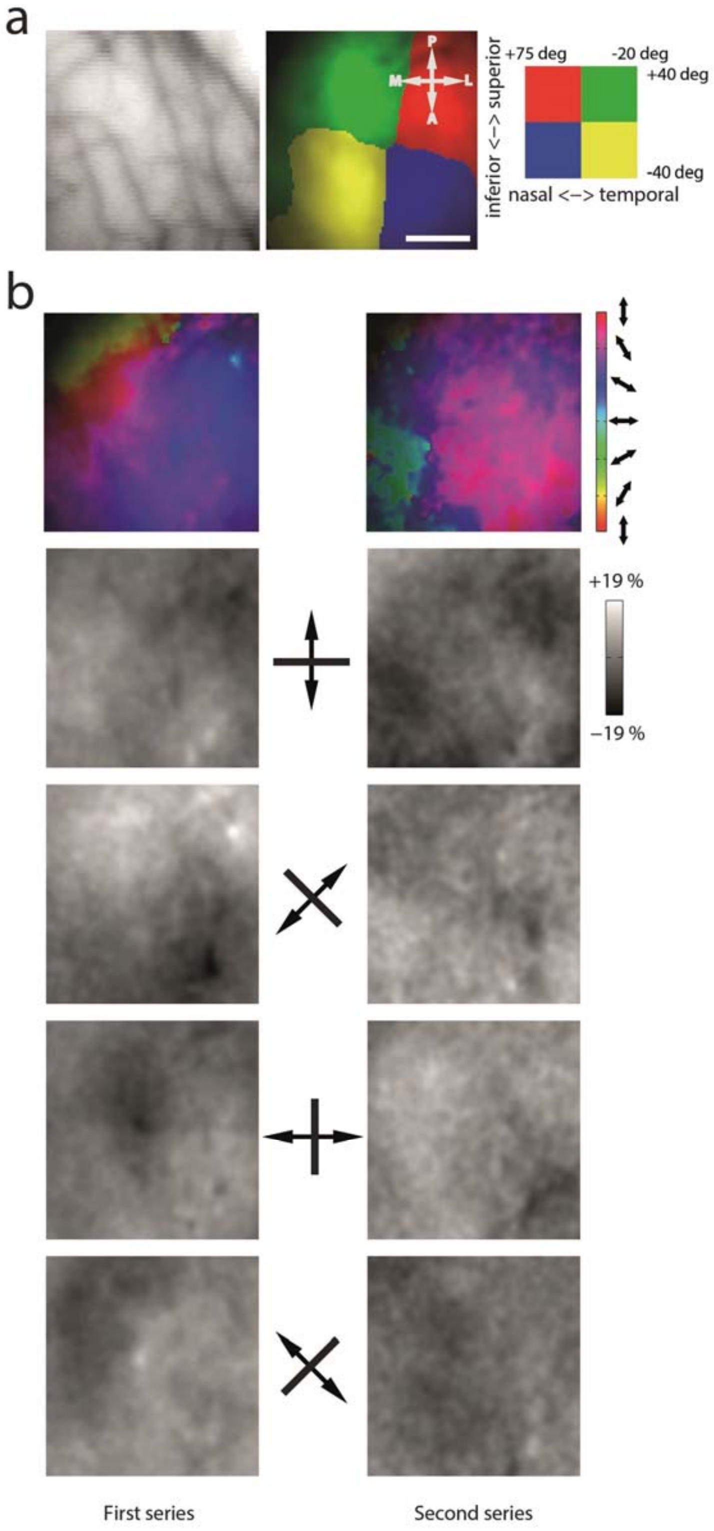

Supplementary Figure 9 Visual cortex lacks a consistent structure for orientation preference. **(a)** Images of the left primary visual cortex (left) and the associated retinotopy (right) (1 mouse). Each pixel is colored with a hue corresponding to the monitor patch to which it gave most response. Pixel saturation scales with response strength. Maximum $\Delta F/F$ response was 3%. Scale bar is 1 mm. **(b)** The difference of the average response for each set of orientations and the average response to all orientations shows regional differences, that are not consistent for the first set of 10 (left) and next set of 10 presentations (right) (1 mouse). Saturation scales with response strength. Maximum $\Delta F/F$ response was 2.5%.

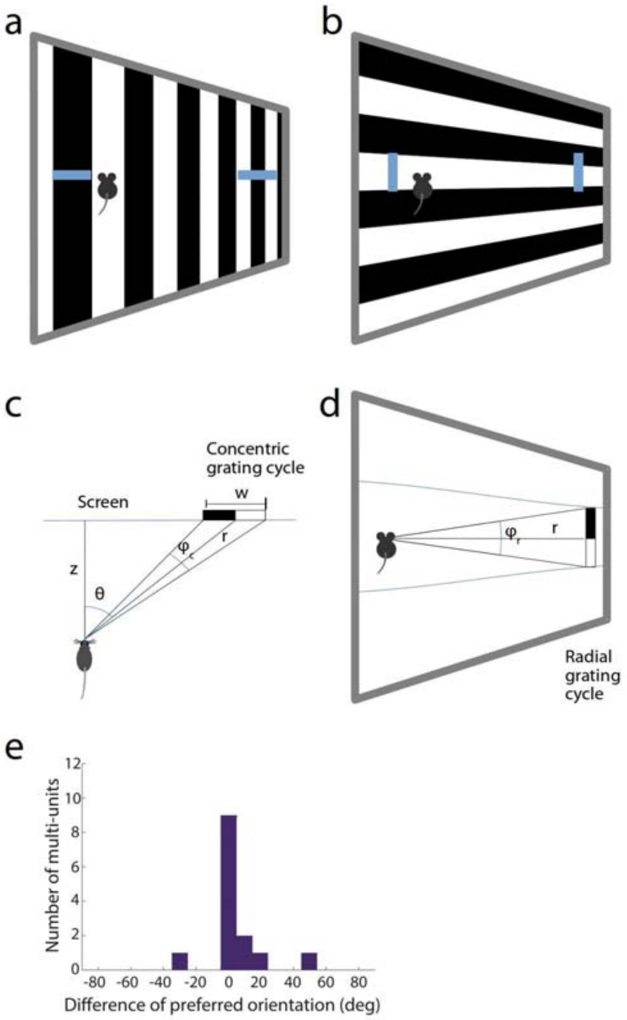

Supplementary Figure 10 Schematic of distortion due to presenting gratings on a flat screen. **(a,b)** When presenting gratings with a fixed physical spacing on a screen, the perceived spatial frequency of the grating closer to the observer is lower than in the periphery. Vertical gratings on the horizontal meridian have a higher spatial frequency in the meridian than horizontal gratings. The blue bars are presented for the reader and have the same physical size on paper. **(c)** The visual angle φ_c of a vertical grating at the horizontal meridian at azimuth θ with cycle width w is $\varphi_c = \arctan(\tan(\theta) + w/(2z)) - \arctan(\tan(\theta) - w/(2z))$. **(d)** The visual angle φ_c of a horizontal grating at the horizontal meridian at azimuth θ with the same width w is $\varphi_r = 2 \arctan(w \cos(\theta) / (2z))$. **(e)** Mean difference between the preferred orientation of units with the standard configuration of the screen (in front of the nose) and with the angled screen (orthogonal to the receptive field location) was not different from 0 ($p = 0.30$, t-test, 14 multi-units, 3 mice).

Supplementary Note 1

For our electrophysiological recordings, stimuli were presented on a large flat screen positioned directly in front of the animal. This induces a distortion in the apparent spatial frequency of the grating stimuli. **Supplementary Figure 10a,b** show that while the horizontal and vertical black and white bars are as wide as the blue bar close to the mouse, they are smaller at the far right further away from the mouse. The horizontal black and white bars are about half the size of the blue bar, while the vertical bars are only about of a third of the size.

To calculate the size of this distortion effect across the visual field, consider **Supplementary Figure 10c,d** which show the placement of the screen, and where we consider a receptive field on the horizontal meridian at azimuth θ. In this figure, z is the shortest distance of the mouse to the screen, w is the fixed width of one cycle of the grating with spatial frequency sf at the shortest distance of the mouse to the screen, such that $\tan(1/(2\,sf)) = w/(2z)$, and φ_c and φ_r are the angular widths of one cycle of the grating at the receptive field location in the concentric and radial orientation, respectively. Using standard trigonometry, we find that for the concentric case, the angular width is given by

$$\varphi_c = \arctan(\tan(\theta) + w/(2z)) - \arctan(\tan(\theta) - w/(2z))$$
$$= \arctan(\tan(\theta) + \tan(1/(2sf))) - \arctan(\tan(\theta) - \tan(1/(2sf)))$$

while for the radial grating the angular width is

$$\varphi_r = 2\arctan(w\cos(\theta) / (2z))$$
$$= 2\arctan(\cos(\theta)\tan(1/(2sf)))$$

We see that for a given angle θ the angular widths are thus independent of the distance of the screen, and are only dependent on the screen placement and the spatial frequency.

The effect of the distortion is that the spatial frequency of a fixed-width grating will depend not only on the location where it is shown but also on its orientation as φ_c and φ_r are not given by the same function. A cell with a unoriented centre-surround receptive field out of the center of the visual field will have a certain preferred spatial frequency. When a vertical grating is shown at this preferred spatial frequency, the horizontal grating with the same physical width will be suboptimal. For a grating of a fixed width, the responses of this neuron will thus be orientation-sensitive. The amount of sensitivity will depend on the specific receptive field location and organization of the cell and on the spatial frequency of the presented grating.

The base spatial frequency which was used for most of our recordings was 0.05 cycles per degree (cpd). Let us consider what this would mean for a typical example receptive field location from **Figure 4h** at about 20 degrees lateral on the horizontal meridian. We find that the angular width of a concentric grating is $\varphi_c = 17.8$ degrees, and for the radial grating $\varphi_r = 18.8$ degrees. The concentric and radial spatial frequencies at this position are then 1/17.8 = 0.056 cpd and 0.053 cpd. This difference of 5% is unlikely to have much influence on the orientation tuning of the neuron, because spatial frequency tuning is usually at least 2 octaves broad (at least in visual cortex)[1].
Further out, the distortion becomes much stronger. At 50 degrees for example, φ_c has shrunk to 8.4 degrees, and φ_r to 12.9 degrees, corresponding to a 35% difference in local spatial frequency. However, at this eccentricity the minimum of these two spatial frequencies already corresponds to 0.08 cpd. Most of the sSC neurons have a preferred spatial frequency of 0.08 cpd or lower[2] and would thus show less response to the concentric grating with the higher spatial frequency, if they were untuned. Therefore, just based on theoretical grounds, it is unlikely that the distortion underlies the observed concentric bias. Experimental evidence against the hypothesis that the concentric bias was due to the distortion is presented in **Supplemental Figure 10e.**

Supplementary References

1. Niell, C.M. & Stryker, M.P. Highly selective receptive fields in mouse visual cortex. J Neurosci. 28, 7520-36 (2008).
2. Wang, L., Sarnaik, R., Rangarajan, K., Liu, X. & Cang, J. Visual receptive field properties of neurons in the superficial superior colliculus of the mouse. J Neurosci. 30, 16573-84 (2010).
